# Verbal, Visual, and Intermediary Support for Child Witnesses with Autism During Investigative Interviews

**DOI:** 10.1007/s10803-017-3142-0

**Published:** 2017-05-13

**Authors:** Lucy A. Henry, Laura Crane, Gilly Nash, Zoe Hobson, Mimi Kirke-Smith, Rachel Wilcock

**Affiliations:** 1 Division and Language and Communication Science, City, University of London, 10 Northampton Square, London, EC1V 0HB UK; 2 Department of Psychology, Goldsmiths, University of London, New Cross, London, SE14 6NW UK; 3 Department of Psychology, University of Winchester, Winchester, SO22 4NR UK; 4 Institute of Education, University College London, London, UK; 5 Metropolitan Police Service, London, UK; 6 West Heath School, Ashgrove Road, Sevenoaks, Kent UK

**Keywords:** Autism, Investigative interviews, Eyewitness memory, Verbal labels, Sketch reinstatement of context, Registered intermediaries

## Abstract

Three promising investigative interview interventions were assessed in 270 children (age 6–11 years): 71 with autism spectrum disorder (ASD) and 199 who were typically developing (TD). Children received ‘Verbal Labels’, ‘Sketch Reinstatement of Context’ or ‘Registered Intermediary’ interviews designed to improve interview performance without decreasing accuracy. Children with ASD showed no increases in the number of correct details recalled for any of the three interview types (compared to a Best-Practice police interview), whereas TD children showed significant improvements in the Registered Intermediary and Verbal Labels interviews. Findings suggested that children with ASD can perform as well as TD children in certain types of investigative interviews, but some expected benefits (e.g., of Registered Intermediaries) were not apparent in this study.

## Introduction

The aim of the current study was to explore whether three promising investigative interview interventions (one practical and two derived from theory) would increase the amount of information recalled about a witnessed event (without hampering accuracy) by child witnesses with and without autism spectrum disorder (ASD). All three interventions were compared to a Best-Practice interview, i.e. an interview carried out according to usual police best-practice in England and Wales, in large samples of children with and without ASD. This study is the first of its kind to explore the usefulness of several different investigative interview interventions in children with ASD.

The context for the study derives from reports of poor memory for events in children with ASD (e.g., Bruck et al. 2007; Goddard et al. 2014; Millward et al. 2000). These findings, coupled with general impairments in social interaction and communication (American Psychiatric Association 2013), may lead to concerns about the reliability of this group as eyewitnesses (McCrory et al. 2007). This is worrying, because eyewitnesses provide key investigative leads, such as suggested lines of enquiry and the identification of possible suspects (Kebbell and Milne 1998). In addition, strength of evidence has been associated with the confessions of guilty suspects (Wells et al. 2006). Finally, although it is difficult to provide estimates of autistic involvement in the criminal justice system (e.g., King and Murphy 2014), individuals with ASD are at increased risk of violence, victimisation and abuse (Petersilia 2001) and may, therefore, be more likely to encounter the criminal justice system.

Experimental studies of eyewitness memory have demonstrated that although children with ASD are as accurate as typically developing (TD) children (i.e., they do not make more errors as a proportion of their total recall), their free recall contains fewer items of correct information (Bruck et al. 2007; McCrory et al. 2007). In addition, children with ASD are no more suggestible than their TD counterparts, and are no more likely to confabulate (Bruck et al. 2007; McCrory et al. 2007). Overall, these studies suggest that children with ASD *can* be reliable witnesses, but that they may provide less information than TD children. Intuitively, employing structured, closed questions would seem appropriate for children with ASD, but Bruck et al. (2007) found this to magnify error rates. It is, therefore, important to develop effective strategies to *increase* the amount of information recalled by child witnesses with ASD, without a corresponding *decrease* in the accuracy of that information. In the current research, we explored the efficacy of two simple and easily implemented theory-based interventions (‘Verbal Labels’ and ‘Sketch Reinstatement of Context’), and one practical intervention (‘Registered Intermediary’) with strong anecdotal (but not empirical) support. All of the interventions can be used to complement and extend a Best-Practice police interview, and they were designed to improve recall at investigative interview without a concomitant decrease in accuracy.

### Verbal Labels

The Verbal Labels procedure (Brown and Pipe 2003) relies on interviewers providing children with four additional *verbal* prompts concerning key aspects of the event (e.g., perpetrators, setting, actions, and conversation). Children first free recall details of the event, and then go on to recall further information about the different *categories* of the event, via verbal prompts from the interviewer. This procedure is straightforward to use, and is argued to facilitate and enhance memory performance by providing relevant external retrieval cues to elicit key information, structured in terms of what is important to convey about a past event (Chae et al. 2014).

In TD children, the Verbal Labels technique increases the amount of information reported by 3- to 5-year-olds (Chae et al. 2014; Kulkofsky 2010) and 6- to 8-year-olds (Brown and Pipe 2003). However, decreases in accuracy have been reported in preschool samples (Chae et al. 2014; Kulkofsky 2010), perhaps reflecting cognitive and social immaturity (i.e., not understanding the requirement to take advantage of verbal cues by providing only accurate information). Potentially beneficial effects have, however, been reported in vulnerable groups (e.g., children with low language abilities; Kulkofsky 2010). The use of Verbal Labels as a ‘scaffolding’ technique for children with ASD is in accordance with the Task Support Hypothesis (Bowler and Gaigg 2008), which argues that providing support at recall improves memory performance in individuals with ASD. It also relates to empirical research showing that children with ASD tend to focus on irrelevant aspects of the environment (Klin et al. 2003), and produce limited narratives during free recall (Bruck et al. 2007; McCrory et al. 2007).

### Sketch Reinstatement of Context (Sketch-RC)

As well as supporting children to identify which aspects of an event need reporting, it is important to provide an effective means by which children can report what they have observed. Jack et al. (2015) suggest a number of practical and theoretical reasons why drawing may be effective in increasing correct recall: it may keep a child more engaged during the interview; ease a potentially uncomfortable interaction with an unfamiliar adult; act as a retrieval cue, which is likely to be accurate because it is self-generated; and act as mental reinstatement of context (MRC, Smith 1979). MRC draws on Tulving and Thompson’s (1973) encoding specificity principle that successful retrieval depends on the similarity between the target memory trace and the retrieval environment. MRC is believed to be one of the most effective components of the Enhanced Cognitive Interview (ECI; Memon et al. 1997), the current police interviewing technique used with adults in England and Wales (Dando et al. 2008). However, police officers do not always use MRC instructions (Dando et al. 2008, 2009a), possibly because they are too time consuming. Further, cognitive interviews show little promise as successful interview interventions for adults with ASD (Maras and Bowler 2010). Therefore, in the current study, simpler reinstatement of context techniques based around drawing were the focus of interest.

Dando et al. (2009b) developed the *Sketch Reinstatement of Context* (Sketch-RC) as a succinct, uncomplicated and easy to implement drawing technique that can be used to aid the recall of an event. Witnesses draw a detailed sketch of whatever they believe will help them to remember the event, including as much detail as they wish. As they draw, they are asked to describe to the interviewer each element of the sketch. The formal interview proceeds after this drawing phase, but the drawing remains available for the witness. Compared with the ECI (which contains more complex MRC instructions), the Sketch-RC interview is as effective in adult witnesses, and does not significantly increase the amount of incorrect information recalled (Dando et al. 2009, 2011). More recently, Mattison et al. (2015, 2016) reported the Sketch-RC technique to be effective at increasing the amount of information children with ASD recall, during both free and probed recall, without a concomitant increase in errors; a promising initial finding needing replication.

### Registered Intermediaries

The justice system in England and Wales provides vulnerable witnesses (including children, as well as individuals with ASD) with the option of a Registered Intermediary (RI). An RI is an impartial, trained professional who facilitates understanding and communication between vulnerable witnesses and members of the justice system. RIs are ‘matched’ with vulnerable witnesses based on the needs of the witness and the skills and expertise of the RI. The role of RIs is wide-ranging, but includes an initial assessment of the witness, and preparation of reports that advise how best to communicate with the witness at all different stages of the criminal investigation (e.g., at interview, identification parade, and trial; Plotnikoff and Woolfson 2015). The use of RIs in England and Wales has steadily increased and there is considerable interest in implementing RI schemes in other countries (Henderson 2015; Plotnikoff and Woolfson 2015). The use of RIs is advised in the case of witnesses with ASD (The Advocate’s Gateway 2015, 2016), and legal professionals have responded favourably to their use with this vulnerable group (e.g., Henderson 2015; Plotnikoff and Woolfson 2007, 2015). However, there has been no empirical evaluation of the effect of RIs on witness performance in either TD or ASD children to date, which represents a significant gap in the literature.

Therefore, the aim of the current study was to explore whether these three promising interventions (Verbal Labels, Sketch-RC, and RI assistance) would increase the amount of information that witnesses (with and without ASD) recall at investigative interview, without a concomitant decrease in accuracy (compared to a ‘Best-Practice’ police interview, in accordance with current police practice in England and Wales). There were three key research questions. First, would any of the three interview types improve performance in children with ASD relative to a Best-Practice police interview? Second, would any of the three interview types improve performance in TD children relative to a Best-Practice police interview? [Note—it is important to answer this question for both samples separately, as those with ASD do not always respond favourably to interview interventions that work for typical individuals (e.g. Maras and Bowler 2010; Maras et al. 2014)]. The final question was, do children with ASD differ from TD children in their performance levels on each interview type? To answer this question, we looked at the full sample to assess group differences and possible interactions between interview comparisons and group. Given previous research evidence, we predicted that the Sketch-RC and Verbal Labels interventions would improve correct performance without compromising accuracy in children with ASD *and* TD children, compared to the Best-Practice police interview. As there was no prior empirical research on the impact of RIs, we tentatively predicted that the RI intervention would, similarly, improve performance in both groups. We also predicted, based on previous findings, that children with ASD would recall fewer details about the event than TD children for all interview types.

## Method

### Participants

To maximise the representativeness of the samples in this study, all recruited participants were included, with minor exceptions described below. The ASD sample comprised 71 children (62 boys, 9 girls) between 6 years 4 months and 11 years 10 months (mean = 9 years 4 months), all with a formal diagnosis of ASD from a clinical professional (obtained independently of the research study and confirmed by the parents and/or the school). Although 72 ASD children were initially recruited, one was excluded because of a full-scale IQ in the intellectual disability range (IQ < 70). The TD sample comprised 199 children (98 boys, 101 girls) between 6 years 7 months and 11 years 3 months (mean = 8 years 7 months). Although 202 children were initially recruited for the TD sample, one was not suitable for this group (a full-scale IQ in the intellectual disability range); and two were excluded because they were unavailable for the investigative interview. To confirm the diagnostic status of the participants, parents were asked to complete the Social Communication Questionnaire (Rutter et al. 2003). Completed questionnaires were received for 153 TD (76.9% of the TD sample) and 49 ASD (69.0% of the ASD sample) children. SCQ scores of the ASD group (mean = 20.04, SD = 6.76) were significantly higher than those of the TD group (mean = 5.21, SD = 4.32), *t*(200) = 18.00, *p* < .001. Data from both samples were collected between April 2013 and January 2016 and all participants attended mainstream or special schools in London and the South of England.

Participants were semi-randomly allocated to one of four interview conditions after receiving their Brief Interviews (see ''Materials and Procedure'' section). Strict random allocation was impossible due to practical issues, schools, and the need to test all children in the RI condition last (to prevent cross-fertilisation to our interviewers). Tables 1 and 2 include details about age, IQ, language, memory and attention variables that were assessed for each sample, and also Brief Interview performance. Each interview condition is presented separately, together with significance tests to indicate whether performance on each of these variables differed across interview conditions. Where differences were found, variables were controlled (where relevant).

**Table 1:** Mean (SD) scores on cognitive variables for children with ASD in each interview condition

Variables	Best-practice (n = 18)	Verbal labels (n = 18)	Sketch-RC (n = 18)	Registered intermediary (n = 17)	Group differences (in bold)
Age	9 years 9 months (19 m)	8 years 11 months (17 m)	8 years 11 months (19 m)	9 years 7 m (14 m)	*F*(3,67) = 1.59, *p* = .20
WASI-II^a^	94.4 (14.2)	102.8 (12.9)	100.4 (14.2)	93.4 (20.0)	*F*(3,67) = 1.53, *p* = .22
TOMAL2 composite^a^	97.1 (17.7)	100.3 (16.0)	95.4 (20.4)	88.7 (25.1)	*F*(3,64) = 0.97, *p* = .41
TOMAL2 Verbal^a^	100.5 (19.8)	107.0 (21.9)	105.8 (15.8)	90.8 (26.6)	*F*(3,65) = 2.05, *p* = .12
TOMAL2 Non-verbal^a^	94.7 (17.8)	93.9 (12.6)	86.2 (25.7)	89.9 (18.7)	*F*(3,64) = 0.71, *p* = .55
BPVS-3^a^	76.5 (8.2)	87.3 (16.0)	91.9 (16.4)	84.3 (19.4)	*F **(3,67)** = **3.16**, ***p*** = .**03** **SRC** > **BP**
ELT-2 sequencing^a^	101.7 (13.8)	102.6 (16.1)	104.8 (8.5)	93.1 (16.5)	*F*(3,65) = 2.23, *p* = .09
ELT-2 grammar & syntax^a^	96.7 (11.6)	101.1 (17.0)	96.3 (14.0)	90.5 (12.8)	*F*(3,60) = 1.43, *p* = .24
CELF-4 recalling sentences^b^	6.4 (3.1)	8.8 (4.7)	8.2 (3.3)	5.4 (4.1)	*F **(3,65)** = **2.80**, ***p*** = .**047** **No sig. diffs** ^**c**^
CELF-4 formulated sentences^b^	5.7 (3.0)	5.9 (3.8)	5.4 (3.4)	4.3 (3.2)	*F*(3,62) = 0.71, *p* = .55
TEA-Ch sky search^b^	9.1 (3.5)	7.1 (3.6)	8.2 (3.8)	7.7 (4.4)	*F*(3,66) = 0.83, *p* = .48
TEA-Ch Score!^b^	7.3 (4.9)	8.5 (3.7)	7.9 (4.3)	6.5 (3.5)	*F*(3,66) = 0.76, *p* = .52
TEA-Ch Dual Task^b^	3.4 (2.9)	7.3 (3.7)	3.8 (3.4)	2.6 (3.3)	**F **(3,65)** = **6.68**, ***p*** = .**001** **VL** > **BP** = **SRC** = **RI**
Brief Interview total correct	23.0 (11.4)	23.5 (11.9)	28.2 (20.1)	22.2 (15.9)	*F*(3,67) = 0.55, *p* = .65

*p < .05, **p < .01

^a^Standardised scores (mean 100, SD 15)

^b^Scaled scores (mean 10 SD 3)

^c^For paired comparisons after Bonferroni corrections

**Table 2:** Mean (SD) scores on cognitive variables for TD children in each interview condition

Variables	Best-practice (n = 75)	Verbal labels (n = 44)	Sketch-RC (n = 42)	Registered intermediary (n = 38)	Group differences (in bold)
Age	8 years 8 months (14 m)	8 years 4 months (13 m)	8 years 3 months (15 m)	9 years 2 months (16 m)	*F(**3,195**) = **5.04**, ***p*** = **.002** **RI** > **VL**, **RI** > **SRC**
WASI-II^a^	108.7 (13.7)	105.8 (12.0)	109.5 (13.5)	102.5 (14.3)	*F*(3,195) = 2.42, *p* = .07
TOMAL2 composite^a^	112.8 (15.9)	112.3 (13.8)	112.0 (12.6)	110.1 (16.4)	*F*(3,195) = 0.29, *p* = .83
TOMAL2 verbal^a^	113.2 (16.9)	113.6 (14.7)	111.0 (13.8)	106.5 (16.6)	*F*(3,195) = 1.82, *p* = .15
TOMAL2 non-verbal^a^	109.5 (17.6)	108.1 (15.6)	110.2 (14.9)	111.6 (20.0)	*F*(3,195) = 0.30, *p* = .83
BPVS-3^a^	94.4 (13.4)	93.9 (12.9)	94.6 (13.9)	87.9 (14.9)	*F*(3,195) = 2.26, *p* = .08
ELT-2 sequencing^a^	109.4 (9.1)	107.5 (9.2)	111.6 (8.5)	109.4 (6.5)	*F*(3,195) = 1.73, *p* = .17
ELT-2 grammar & syntax^a^	106.6 (10.7)	106.5 (10.3)	108.6 (9.1)	103.7 (10.8)	*F*(3,194) = 1.53, *p* = .21
CELF-4 recalling sentences^b^	10.4 (3.2)	11.6 (2.3)	11.2 (2.9)	10.8 (3.1)	*F*(3,195) = 1.80, *p* = .15
CELF-4 Formulated sentences^b^	9.3 (3.3)	10.1 (2.9)	10.5 (2.8)	9.1 (3.2)	*F*(3,195) = 2.12, *p* = .10
TEA-Ch sky search^b^	9.5 (2.5)	9.0 (2.8)	9.0 (2.7)	9.2 (3.3)	*F*(3,195) = 0.35, *p* = .79
TEA-Ch score!^b^	9.0 (3.4)	9.1 (3.4)	9.3 (3.8)	9.3 (3.6)	*F*(3,195) = 0.10, *p* = .96
TEA-Ch dual task^b^	6.6 (3.7)	6.6 (3.6)	6.1 (3.8)	5.4 (3.6)	*F*(3,195) = 1.10, *p* = .35
Brief interview total correct	32.2 (15.4)	35.4 (14.2)	32.9 (15.9)	41.6 (12.6)	*F **(3,195)** = **3.68**, ***p*** = **.013** **RI** > **BP**

*p < .05, **p < .01

^a^Standardised scores (mean 100, SD 15)

^b^Scaled scores (mean 10 SD 3)

## Materials and Procedure

This study was conducted in two phases.

### Phase 1: Staged Event and Evidence Gathering Statements (‘Brief Interviews’)

Children watched a live event during school assembly (or a video of this event) of two actors giving a talk about what school was like a long time ago. Ideally all children would have seen the event live, but this was impractical for many children with ASD. Therefore, we checked that interview performance did not differ depending on live versus video viewing, which it did not,^1^ and data for children who viewed the event live or on video were combined. The talk had educational content (regarding school in Victorian times), but included a minor crime (a theft). Children were randomly assigned to view one of two parallel talks each involving slightly different materials (e.g., Version A involved the theft of a phone, whereas Version B involved the theft of a set of keys) to provide some measure of the generalisability of our findings. As there were no significant differences between the two versions of the event (for the samples separately and combined),^2^ data for the two events were combined.

Towards the end of the talk, the ‘theft’ of the phone or keys was explained as a misunderstanding, to avoid exposing children to stress or anxiety. Staged events are usually followed, somewhat later, by a full evidential interview. However, in real-life, response officers typically question witnesses immediately after the event (referred to here as ‘Brief Interviews’). Thus, all participants witnessed the event and, on the same day, one of seven interviewers (pre- or post-doctoral research assistants) questioned every child individually using the exactly same format: a standard protocol that began with the open question: ‘Tell me what you remember about what you just saw’; and a series of follow-up prompts (who was there? what did they do? what did they look like? when did it happen? where did it happen?) that could be used depending on what was said in response to the initial question.^3^

### Phase 2: Investigative Interviews

One week later, children took part in one of four types of investigative interviews, administered by one of three trained interviewers (post-doctoral research assistants) who attended a 1 week Investigative Interviewing Victim & Witness Training Course (provided by the UK’s Metropolitan Police Service). [Note: There was no effect of interviewer on the total amount of correct information recalled for the combined sample, (*F*(2,267) = 1.44, *p* = .24), and nor were there interviewer effects for the ASD (*F*(2,68) = 1.72, *p* = .19) or TD (*F*(2, 196) = 1.24, *p* = .29) groups separately.]

The Best-Practice police interview was based on Achieving Best Evidence principles (Home Office 2011) and had seven discrete phases: (1) greet and personalise the interview; (2) rapport building (chatting to the child about areas of interest); (3) truth and lies exercise (e.g., determining whether the child correctly responds to a statement along the lines of ‘that lady is wearing a blue jumper’ when it is red); (4) explain the purpose of the interview; (5) free recall (recall attempt 1—‘Tell me everything you can remember about what you saw’); (6) questioning (recall attempt 2—using open questions based upon what the child had already recalled); and (7) closure.

For the Verbal Labels and Sketch-RC interviews, only phase 5 (free recall) was manipulated, to encompass the specific instructions for each intervention. In the Verbal Labels procedure, phase 5 (free recall) was followed by ‘tell me more’ prompts in relation to four key areas (adapted from Brown and Pipe 2003): (1) the people in the event; (2) the setting where the event took place; (3) the objects involved and what happened with them (actions); and (4) what the people said. In the Sketch-RC condition, prior to phase 5 (free recall), witnesses were instructed to think about the event and draw whatever reminded them about it; narrating to the interviewer as they were drawing. When participants had finished their sketch, they were asked to give a free recall account of what happened (as per the Best-Practice police interview), and were told that they could use their drawing to point things out or explain things (if they wished). Interviews focused on verbal evidence that the child provided, however, the total number of items drawn by the children on their Sketch Plans was summed. As these data were skewed, a log transformation was applied prior to analyses. Whilst ASD children (mean = 10.89, SD = 5.92) drew fewer items in their sketches than TD children (mean = 14.71, SD = 9.73), an independent samples t-test did not find this to be statistically significant, *t*(58) = −0.97, *p* = .33. There was a significant correlation between the total number of items drawn in the sketches and the total number of items recalled at investigative interviews (calculated by summing the total number of correct, incorrect and confabulated items) in the ASD group (*r* = .62, *p* = .006) but not in the TD group (*r* = .22, *p* = .16). A similar relationship was found looking at the total number of correct items recalled only. These results are consistent with those of Mattison et al. (2015).

Children in the RI condition were individually assessed by one of two experienced, practising RIs on one occasion prior to their interview (as advised by Plotnikoff and Woolfson 2015). This assessment included rapport building, an assessment of the child’s ability to talk about past events (unrelated to the event), and a picture story retelling task to check various types of understanding (including: sequencing; time words, such as *first last after before*; estimating the ages of protagonists; responses to wh- questions; and the ability to verbalise a story and comprehend subtle, implied aspects). The RIs also checked the children’s: understanding of prepositions (e.g., *in, on, under, behind*); ability to recognise emotions; and their drawing and describing ability. An assessment of the individual child’s needs regarding additional concrete or visual communication aids (and their ability to use these aids) was also carried out. Based on this assessment, the RI provided written and verbal recommendations to the interviewer for all aspects of the interview: introductions and rapport building (games, pictures, drawing, calming objects); truth and lies (adding an additional truth and lies procedure using a cartoon story); explaining the rules of the interview supported by picture cards (e.g., only tell what really happened, no making things up or pretending); using visual or concrete aids to support questions and facilitate responses (e.g., paper and pens, small figures and furniture); and advice relating to style or type of questioning, including ways to facilitate further explanation and description (e.g., ‘what else happened’, ‘tell me one more thing’). Note that concrete aids were only used to support communication where needed, and were never used for play (see Brown 2011, for discussion).

There was a meeting between the RI and the interviewer before each child’s interview to discuss the recommendations, during which the RIs flagged any individual needs (note that many adaptations suggested by the RIs applied to the majority of TD children, whereas adaptations for children with ASD were more varied). Regarding the interview, the RIs advised the interviewer to follow the protocol for the Best-Practice interview, with some adaptations (e.g., simplifying the verbal instructions given to the children, and recommending the use of visual cues that were provided by the RIs). At all times, the RI was present to facilitate communication between the child and the interviewer. As the interviewer proceeded through the Best-Practice interview protocol, the RI intervened when appropriate to prompt the interviewer in order to facilitate effective communication (verbally or by suggesting the use of suitable props).

There was no significant difference in number of correct details recalled depending on which of our two RIs the children worked with in the ASD sample (n = 7 vs. n = 10) (*t*(15) = 0.10, *p* = .93), however there was a difference for the TD sample (n = 19 vs. n = 19) (*t*(36) = 3.46, *p* = .001): one RI (mean = 62.21, SD = 15.28) had a more beneficial effect on the total amount of correct information recalled than the other (mean = 44.63, SD = 17.75). No RI differences emerged on error scores (incorrect or confabulated details) in either sample.

Each interview was audio-taped, transcribed, and coded for the total number of correct details recalled: e.g., “The man (1) with the blonde hair (1), Alex (1), stole (1) the man (1) with the brown hair’s (1) keys (1)” = 7 units of correct information. We also coded total number of incorrect details and total number of confabulations. Further coding was carried out to classify correct details by type (adapted from Memon et al. 1997) relating to six key areas: people (descriptions of the men giving the talk, e.g., their names, clothing, appearance); setting (descriptions of the environment in which the event took place, or the time it happened); actions (information about what the men did, e.g., holding X, moving Y); conversations (verbatim accounts of what the men said to the children, e.g., “Alex said ‘where’s my phone?’”); objects (i.e., names or descriptions of the items the men had); and other information about the event that we classified as ‘general’ information (e.g., facts about Victorian times that the children were told during the talk, which were not recalled as verbatim conversation items, e.g., “girls did needlework”). Only unique utterances were coded (repeated information was ignored). Ten percent of all transcripts were double-coded and Pearson product-moment correlation coefficients were calculated for both Brief (correct = 0.98, incorrect = 0.88, confabulations = 0.88) and Investigative (correct = 0.92, incorrect = 0.89, confabulations = 0.76) Interviews.

### Control Measures

Several cognitive measures (intelligence, language, memory and attention) were administered to ensure that cognitive skills that may affect eyewitness recall (e.g., Jack et al. 2014) could either be controlled or matched across interview conditions, in order to increase confidence in the findings—i.e. that any differences between interviews could not be attributed to cognitive differences (see Tables 1, 2). There were missing data for a few subtests, but as these data represented such a small proportion of the dataset, values were left as missing for all relevant analyses.

#### Intelligence

Two subscales of the second edition of the Wechsler Abbreviated Scale of Intelligence (WASI-II; Wechsler and Zhou 2011)—Vocabulary and Matrix Reasoning—were used to establish suitability for the study, and to provide a baseline assessment of intellectual ability (administration time 15 min).

#### Language

The British Picture Vocabulary Scale Third Edition (BPVS-3; Dunn et al. 2009) was used as a well-established test of receptive vocabulary (administration time 10–15 min). Two subtests of the Expressive Language Test 2 (ELT-2, Bowers et al. 2010) were used: Sequencing (a test of narrative ability) and Grammar and Syntax (grammatical morphology) (total administration time 15 min). Two subtests of the Clinical Evaluation of Language Fundamentals, 4th edition (CELF-4 UK; Semel et al. 2006) were included: ‘Recalling Sentences’ (assesses the ability to recall a sentence correctly and reflects grammatical understanding), and ‘Formulated Sentences’ (assesses the child’s ability to formulate complete, grammatically correct and meaningful sentences) (total administration time 15–20 min).

#### Memory

Four of the eight core subtests from the Test of Memory and Learning 2 (TOMAL-2; Reynolds and Voress 2007) were used to provide a composite memory measure reflecting both verbal memory (‘Memory for Stories’ and ‘Paired Recall’) and non-verbal memory (‘Facial Memory’ and ‘Visual Sequential Memory’). Subtests reflected memory skills relevant to the witness skills involved in this study (administration time 25 min).

#### Attention

The Test of Everyday Attention for Children (TEA-Ch; Manly et al. 1999) was used to assess attention skills: selective/focused attention (the ‘Sky Search’ subtest); sustained attention (the ‘Score!’ subtest); and sustained-divided attention (the ‘Sky Search Dual Task’ subtest) (total administration time 15 min).

The study was given full ethical approval at the University at which it was carried out. All children had informed, written parental consent and gave their own written and verbal assent to participate. The Brief Interviews took place one week prior to the Investigative Interviews. Cognitive testing took place by the same team of interviewers (to enhance rapport between researchers and children) and was split over several sessions to fit in with school timetables, and to ensure the children remained engaged with the tasks. As part of this project, the children also took part in an identification parade and cross-examination, but these data are not reported here.

## Results

Hierarchical multiple regression was used to: (1) control cognitive variables that differed between interview conditions (Tables 1, 2); and (2) assess differences in performance across interview conditions (steps 1 and 2 of each regression respectively). For all regression analyses, key statistical checks (Durbin-Watson, tolerance and VIF statistics, Cook’s and Mahalanobis distances, standardised DFβs, leverage values, plots of standardised residuals and predicted standardised values, standardised residuals, partial plots) were carried out to ascertain that no individual cases had undue influence on the regressions (Field 2013). For error data, log transformations were performed, and proportion correct data were subject to an arcsine transformation prior to analyses (Cohen and Cohen 1983).

### Research Question 1: Did the Interview Interventions Improve Performance in Children with ASD?

Interview condition differences in performance were assessed for four dependent variables: total correct details; total incorrect details; total confabulations; and proportion of correct details (see Table 3 for mean raw scores). The three variables that differed between interview groups (receptive vocabulary, dual task attention, Recalling Sentences; see Table 1) were initially entered at Step 1 of each regression, but the only variable that ever related to interview performance was receptive vocabulary, therefore, the final models retain only this variable. Three dummy-coded interview condition variables, introduced at Step 2, assessed interview condition differences between the reference condition (Best-Practice interview) and each of the other three interview conditions. Table 4 gives full details of Step 2 from each regression.

**Table 3:** Mean (SD) raw scores for correct, incorrect, confabulated, and proportion accurate details in the investigative interview for children with ASD in each interview condition, as well as numbers of details in the six information categories (people, setting, actions, conversation, objects, general)

Measures	Interview condition			
	Best-practice	Verbal labels	Sketch-RC	Registered intermediary
Correct details	20.06 (15.11)	32.00 (16.56)	27.17 (24.11)	18.47 (16.61)
Incorrect details	2.67 (2.93)	3.11 (3.48)	2.17 (2.20)	2.82 (3.38)
Confabulations	1.50 (2.15)	3.67 (5.02)	3.78 (4.89)	1.94 (2.90)
Proportion of correct details	0.82 (0.12)	0.81 (0.16)	0.78 (0.20)	0.79 (0.20)
People	7.83 (6.11)	11.61 (6.77)	8.61 (6.79)	6.94 (6.71)
Setting	1.11 (1.28)	2.17 (1.76)	1.28 (1.94)	0.41 (0.51)
Actions	4.39 (4.46)	6.22 (4.55)	5.72 (6.70)	3.94 (4.49)
Conversation	0.50 (0.99)	1.94 (2.90)	1.44 (2.01)	0.88 (1.58)
Objects	2.83 (2.66)	5.22 (4.40)	4.89 (4.66)	3.65 (3.72)
General	3.39 (3.98)	4.83 (4.03)	5.22 (5.40)	2.65 (3.46)

**Table 4:** Details of step 2 for regressions predicting investigative interview performance in children with ASD

Investigative interview measures	Total R^2^ accounted for by the model	Change in R^2^ at step 2	β’s receptive vocabulary	β’s best-practice vs. verbal labels	β’s Best-practice vs. sketch-RC	β’s Best-practice vs. RI
Total correct details	0.30***	0.05	0.50***	0.13	−0.04	−0.14
Total incorrect details	0.10	0.02	0.32*	−0.05	−0.19	−0.11
Total confabulations	0.08	0.06	0.07	0.21	0.23	0.02
Proportion of correct details	0.02	0.01	0.12	−0.03	−0.09	−0.04

Receptive vocabulary was entered first, with three dummy-coded interview condition variables entered second. Information includes data for step 2 of the model: total variance accounted for (total *R* ^2^); change in *R* ^2^; and standardised β-values. n = 71

*p < .05, **p < .01, ***p < .001

For total correct details, the full regression model was significant (*F*(4,66) = 7.22, *p* < .001) and accounted for 30.4% of the variance. Introducing the dummy coded interview condition variables at Step 2 resulted in no significant change in *R* ^2^ (5.0%), indicating no significant differences in performance across interview conditions (*F Change* (3,66) = 1.59, *p* = .20). Standardised β-values indicated that receptive vocabulary was significantly related to number of correct details recalled on the Investigative Interview (*p* < .001).

For total incorrect details, the full regression model accounted for 9.7% of the variance and was not significant (*F*(4,66) = 1.77, *p* < .15), although Step 1 of the model was significant and showed an effect for receptive vocabulary (*p* = .02). Introducing dummy coded interview condition variables at Step 2 did not result in a significant change in *R* ^2^ (*F Change* (3,66) = 0.58, *p* = .63, 2.4% of the variance), indicating no significant interview condition differences. For total confabulations, the full regression model accounted for 7.6% of the variance and was not significant (*F*(4,66) = 1.36, *p* = .22). Introducing dummy coded interview condition variables at Step 2 did not result in a significant change in *R* ^2^ (*F Change* (3,66) = 1.30, *p* = .28, 5.5% of the variance), indicating no significant interview condition differences. For proportion of correct details, n = 59 because 12 children (distributed across the four interview conditions) recalled nothing in the Investigative Interviews, therefore no proportion correct values could be calculated. The full regression model accounted for 1.5% of the variance and was not significant (*F*(4,54) = 0.21, *p* = .93). Introducing dummy coded interview condition variables at Step 2 did not result in a significant change in *R* ^2^ (*F Change* (3,54) = 0.10, *p* = .96), indicating no significant interview condition differences.

Correct details were also coded for type of information recalled (people, setting, actions, conversation, objects, general—see Table 3 for mean raw scores), and similar regressions were used to assess potential differences between interview conditions in each of these sub-categories. Alpha was set at *p* < .008 after Bonferroni corrections based on six additional regressions. Log transformations were applied for setting, actions, conversation and general details. The overall regression models were significant for all types of details except conversation details [people (*F*(4,66) = 3.43, *p* = .01), setting (*F*(4,66) = 6.06, *p* < .001), actions (*F*(4,66) = 5.51, *p* = .001), objects (*F*(4,66) = 4.95, *p* = .002) and general (*F*(4,66) = 7.43, *p* < .001); the regression model missed significance for conversation details (*F*(4,66) = 2.99, *p* = .02)].

Of particular interest was whether there were interview condition differences for any of these types of details, i.e. significant changes in *R* ^2^ at Step 2 of the models. In fact, there were no significant interview condition differences for people, action, conversation, object or general details, but there was a difference for setting details (*F Change* at step 2 (3,66) = 5.17, *p* = .003). Inspection of the standardised β-values indicated that the contrast between the Best-Practice interview and the RI interview was marginally significant (*p* = .03): children in the RI condition tended to recall fewer setting details (although note that numbers of setting details recalled were small across all interview conditions). In terms of other predictors, receptive vocabulary was a significant predictor of all types of details except conversation details [people (β = 0.34, *p* = .006), setting (β = 0.33, *p* = .005), actions (β = 0.47, *p* < .001), object (β = 0.44, *p* < .001), and general (β = 0.52, *p* < .001); although receptive vocabulary also related to conversation details (β = 0.34, *p* = .006), this result cannot be interpreted as the overall regression model was non-significant].

#### Summary

For children with ASD, none of the interview interventions significantly improved overall number of correct details recalled, type of details recalled, or error rates compared to a Best-Practice interview. There was a marginally significant tendency for those in the RI condition to recall fewer correct setting details.

### Research Question 2: Did the Interview Interventions Improve Performance in TD Children?

Similar regressions were carried out for correct, incorrect, confabulated and proportion correct details for the TD group (see Table 5 for mean raw scores). Variables that differed significantly between interview conditions were included at Step 1 to control for their effects (these were age and Brief Interview total correct—see Table 2): we also included IQ, which showed a marginally significant interview group difference. Table 6 gives details about step 2 of each regression.

**Table 5:** Mean (SD) raw scores for correct, incorrect, confabulated, and proportion accurate details in the investigative interview for TD children in each interview condition, as well as numbers of details in the six information categories (people, setting, actions, conversation, objects, general)

Measures	Interview condition			
	Best-practice	Verbal labels	Sketch-RC	Registered intermediary
Correct details	30.04 (18.03)	37.59 (15.31)	32.40 (19.15)	53.92 (18.85)
Incorrect details	3.35 (2.83)	4.18 (3.47)	3.48 (2.89)	5.47 (3.20)
Confabulations	2.73 (4.07)	5.52 (8.51)	5.14 (6.80)	3.84 (4.61)
Proportion of correct details	0.84 (0.10)	0.79 (0.18)	0.77 (0.18)	0.84 (0.11)
People	10.57 (7.19)	11.80 (6.63)	9.64 (7.70)	18.39 (7.36)
Setting	1.03 (1.20)	2.07 (1.19)	1.05 (1.40)	1.66 (1.48)
Actions	5.51 (4.58)	6.34 (4.19)	5.48 (4.71)	10.74 (6.00)
Conversation	1.27 (1.80)	1.07 (1.45)	1.12 (2.18)	2.29 (2.73)
Objects	4.71 (3.59)	7.66 (3.87)	6.52 (4.92)	10.29 (5.64)
General	6.96 (4.66)	8.61 (4.76)	8.60 (4.05)	10.58 (4.59)

**Table 6:** Details of step 2 for regressions predicting investigative interview performance in TD children

Investigative interview measures	Total R^2^ accounted for by the model	Change in R^2^ at step 2	β’s Age	β’s brief total correct	β’s IQ	β’s Best-practice vs. verbal labels	β’s Best-practice vs. sketch-RC	β’s Best-practice vs. RI
Total correct details	0.56***	0.11***	0.27***	0.38***	0.18***	0.18**	0.08	0.38***
Total incorrect details	0.22***	0.02	0.08	0.37***	0.00	0.07	0.02	0.16*
Total confabulations	0.07*	0.04*	0.20**	0.00	0.05	0.19*	0.19*	0.07
Proportion of correct details	0.05	0.03	−0.07	0.08	0.09	−0.13	−0.17*	0.02

Age, Brief Interview total correct and IQ were entered first, with three dummy-coded interview condition variables entered second. Information includes data for step 2 of the model: total variance accounted for (total *R* ^2^); change in *R* ^2^; and standardised β-values. n = 199

*p < .05, **p < .01, ***p < .001

For total correct details, the full regression model was significant (*F*(6, 192) = 40.62, *p* < .001). Introducing the dummy coded interview condition variables at Step 2 of the regression resulted in a significant change in *R* ^2^, indicating significant differences in performance across interview types (*F Change* (3, 192) = 15.86, *p* < .001). Inspection of the standardised β-values at Step 2 showed that children receiving the RI (*p* < .001) and Verbal Labels (*p* = .001) interviews recalled significantly more information than children receiving the Best-Practice interview. After accounting for the other variables, children in the RI interview recalled 18.96 more items of correct information than children in the Best-Practice interview (95% CI 13.43–24.49 items); and children in the Verbal Labels condition recalled 8.47 more items of correct information than children receiving a Best-Practice interview (95% CI 3.35–13.59 items). Age, Brief Interview total correct and IQ were also significantly related to Investigative Interview performance (*p*s < 0.001). The full model accounted for 55.9% of the variance, and the change in *R*² at Step 2 of the model was 10.9% (*p* < .001).

For total incorrect details, the full regression model was significant (*F*(6, 192) = 9.06, *p* < .001). Introducing the dummy coded interview condition variables at Step 2 did not result in a significant change in *R* ^2^ (*F Change* (3, 192) = 1.75, *p* = .16), indicating no significant interview condition differences. Inspection of the standardised β-values showed that Brief interview total correct score (*p* < .001) was significantly related to total incorrect details. Note: although the β-values showed a significant contrast between the Best-Practice and RI conditions, *p* = .03, the lack of an overall *R* ^2^ change at step 2 of the model means this result cannot be interpreted. The full model accounted for 22.0% of the variance, and the change in *R* ^2^ at Step 2 of the model was 2.1%.

For total confabulations, the full regression model was significant (*F*(6, 192) = 2.54, *p* = .02). Introducing the dummy coded interview condition variables at Step 2 resulted in a significant change in *R* ^2^ (*F Change* (3, 192) = 2.82, *p* = .04). Standardised β-values at Step 2 revealed that children in the Verbal Labels (*p* = .02) and Sketch-RC (*p* = .018) conditions made more confabulations than children in the Best-Practice condition. Age was significantly related to total confabulations (*p* = .01). The full model accounted for 7.4% of the variance, and the change in *R* ^2^ at Step 2 of the model was 4.1%.

For proportion of correct details, n = 193 as six children did not recall any correct details in the investigative interview (five in the Best-Practice interview, one in the Verbal Labels interview). The full regression model was not significant (*F*(6, 186) = 1.54, *p* = .17). Introducing the dummy coded interview condition variables at Step 2 of the regression did not result in a significant change in *R* ^2^ (*F Change* (3, 186) = 2.18, *p* = .09), indicating no significant interview condition differences. Note: although the contrast between the Best-Practice and Sketch-RC interviews was significant (*p* = .04), this cannot be interpreted as the overall regression model was not significant. The full model accounted for 4.7% of the variance, and the change in *R* ^2^ at Step 2 of the model was 3.3%.

Correct details were also coded for type of information recalled (people, setting, actions, conversation, objects, general—see Table 5 for mean raw scores), and similar regressions were used to assess interview condition differences in each of these sub-categories. Alpha was set at *p* < .008 after Bonferroni corrections based on six regressions. Log transformations were applied to setting and conversation data. The regression models were significant for all types of details [people (*F*(6, 192) = 19.98, *p* < .001); setting (*F*(6, 192) = 10.90, *p* < .001); actions (*F*(6, 192) = 21.61, *p* < .001); objects (*F*(6, 192) = 21.88, *p* < .001); conversation (*F*(6, 192) = 7.92, *p* < .001); and general (*F*(6, 192) = 16.69, *p* < .001)]. Of particular interest was whether there were interview condition differences for any of the types of details, i.e. significant changes in *R* ^2^ at Step 2 of the models. Such differences were found for all types of correct details except conversation details [people (*F Change* (3, 192) = 8.81, *p* < .001); setting (*F Change* (3, 192) = 10.57, *p* < .001); actions (*F Change* (3, 192) = 7.90, *p* < .001); objects (*F Change* (3, 192) = 14.33, *p* < .001); and general (*F Change* (3, 192) = 5.76, *p* = .001); *R* ^2^ change for conversation details was not significant (*F Change* (3, 192) = 2.25, *p* = .08].

In order to interpret these interview condition differences, the β-values were inspected. These indicated that the following interview condition differences were present: (1) Children in the RI interview recalled significantly more details about people (β = 0.31, *p* < .001) and actions (β = 0.31, *p* < .001) than children in the Best-Practice interview. (2) Children in the Verbal Labels interview recalled significantly more details about setting than children in the Best-Practice interview (β = 0.37, *p* < .001). (3) Children in all three interview intervention conditions (Verbal Labels, Sketch-RC and RI) recalled significantly more details about objects (β’s = 0.28, *p* < .001; 0.18, *p* = .004; and 0.39, *p* < .001 respectively) and general information (β’s = 0.19, *p* = .006; 0.18, *p* = .008; and 0.25, *p* < .001 respectively) than children in the Best-Practice interview. In terms of other predictors of correct recall for types of details, Brief Interview total correct was a significant predictor for all types of details except setting [person (β = 0.38, *p* < .001), actions (β = 0.25, *p* < .001), conversation (β = 0.41, *p* < .001), objects (β = 0.25, *p* < .001), and general (β = 0.23, *p* = .001)]. Age was related to correct recall of all types of details except person and conversation [setting (β = 0.20, *p* = .004), actions (β = 0.33, *p* < .001), objects (β = 0.25, *p* < .001), and general (β = 0.28, *p* < .001)]. Finally, IQ related to performance on two types of details [objects (β = 18, *p* = .003) and general (β = 0.23, *p* < .001)].

#### Summary

For TD children, the RI and Verbal Labels interviews increased the number of correct details recalled compared to a Best-Practice interview. RI interviews showed the greater increase, without affecting error rates. In contrast, the Verbal Labels interview increased the number of confabulations. In terms of types of details recalled, all interview interventions led to at least some improvements: RI interviews increased the number of people, actions, objects and general details recalled; Verbal Labels interviews increased the number of setting, objects and general details recalled; and Sketch-RC interviews increased the numbers of objects and general details recalled.

### Research Questions 3 and 4: Were There ASD/TD Group Differences in Performance on the Investigative Interview, and did the Interview Interventions Affect Performance Differently in the Two Samples of Children?

Hierarchical multiple regression was used to test these two research questions by including all participants in the same regressions. Step 1 reflected the variables included in the previous regressions, and for this we merged the background variables that differed between interview conditions in the ASD and TD samples (age, receptive vocabulary, Brief Interview correct details—we did not include IQ as this was strongly related to receptive vocabulary); plus the dummy-coded interview condition variables (with the Best-Practice interview acting as the reference condition in each case). To test for overall group differences in performance (Research question 3), group was entered at Step 2 of the model (ASD versus TD). To test whether group interacted with interview condition (Research question 4), three interaction variables (Jaccard et al. 1990) were entered at Step 3 (those between group and each of the dummy-coded interview condition variables).

For correct details, one multivariate outlier was identified, but as removing this case made no difference to the results, it was retained. The overall regression model for correct details was significant (*F*(10, 259) = 35.81, *p* < .001, accounting for 58% of the variance). The three control variables were significant at step 1 and remained so by step 3 (β age = 0.21, *p* < .001; β Brief Interview total correct = 0.46, *p* < .001; β receptive vocabulary = 0.19, *p* < .001). The significant interview condition contrasts exactly corresponded to those found for the TD sample (i.e. RI > Best-Practice; Verbal Labels > Best-Practice; Sketch-RC = Best-Practice)—these results were not surprising as TD children formed the majority of the combined sample.

At step 2, there was a significant change in *R* ^2^ (*F Change* (1,262) = 9.71, p = .002, 1.7% of the variance) with the entry of group (β = 0.15, *p* = .002), which initially suggested an overall ASD versus TD group difference in interview performance. However, there was also a significant change in *R* ^2^ (*F Change* (3, 259) = 8.65, p < .001, 4.2% of the variance) with the entry of the interaction terms at step 3, and, critically, at this final step the group effect became non-significant (β = 0.07, *p* = .38). This means that overall ASD versus TD group differences were different for different interview condition comparisons. The term reflecting the RI versus Best-Practice interview by group interaction was significant (β = 0.36, *p* < .001), which confirmed the separate sample analyses reported earlier showing that whilst RIs improved recall of correct details compared to a Best-Practice interview in TD children, this beneficial effect was not observed for children with ASD. The comparison between the Best-Practice interview and, respectively the Verbal Labels and Sketch-RC interviews, did not interact with group (β’s = –0.08 and 0.01, *p*s = 0.38 and 0.91), indicating that ASD/TD group differences were not apparent for either of these interview contrasts: performance levels were no different between ASD and TD children.

For incorrect details, although the full regression model was significant (*F*(10, 259) = 7.55, *p* < .001), the only significant β-value at step 3 was for Brief Interview total correct (β = 0.35, *p* < .001). For confabulations, the full regression model was again significant (*F*(10, 259) = 1.99, *p* = .03), but no individual β-values were significant at step 3. The full regression model for proportion of accurate details was not significant. Hence, there were no group differences or interactions for error scores or proportion of accurate details.

#### Summary

Recall of correct details was significantly higher in the RI than the Best-Practice interview for TD children, but the beneficial effect of RIs was not observed for children with ASD. The other interview contrasts (Verbal Labels with Best-Practice; Sketch-RC with Best-Practice) did not interact with group: this indicated that children with ASD and TD performed at the same level on these interviews and there were no significant group differences in their relative effects.

## Discussion

The present study evaluated the utility of three promising interview techniques (Verbal Labels, Sketch-RC, and RI assistance) predicted to improve recall at Investigative Interview in 6–11-year-old children with and without ASD. For children with ASD, contrary to predictions, none of the interview interventions increased the number of correct details recalled about a witnessed event; although neither did any of these interview interventions hamper their performance. By contrast, for TD children, significantly better overall performance (compared to a Best-Practice police interview) was found for the RI interview: this increased the recall of correct details by a substantial amount, whilst not significantly increasing the numbers of incorrect details or confabulations. TD children who received a Verbal Labels intervention also recalled more correct details without an increase in the number of incorrect details, but there was some evidence for higher rates of confabulations.

Perhaps the most surprising finding was that the RI intervention did not improve the volume of correct information recalled by children with ASD, whereas it was highly effective for the TD children. The most effective interventions for children with ASD tend to be individualised—considering how ASD uniquely affects each child (Hurth et al. 1999)—and the RI intervention is based upon this principle. For example, RIs conduct detailed assessments with each witness to determine their specific communication needs, they liaise with people who best know the witness (e.g., parents, teachers), and they make recommendations about how each witness can give their best evidence (if at all). Considering why the RI intervention was not effective for ASD children, it is possible that the beneficial effects of RIs for this group are not in terms of increasing the volume of recall (as assessed in the current study), but are in relation to other issues. RIs have a broad remit, including but not limited to: informing the police and the court about ASD and how it affects individual witnesses; building rapport through individualised introductory letters, social stories, and meetings; advising and assisting the police with familiarising the witness with the investigative process; and making recommendations about the interview environment. These varied and important aspects of the RI role were not addressed in the current study and were further constrained by the fact that, for ethical purposes, all researchers involved had knowledge and experience of ASD and all children were interviewed and assessed in familiar surroundings. In some cases, the presence of an RI can be the critical factor in determining whether a witness can be called to give evidence at all (Plotnikoff and Woolfson 2015), so further work should systematically evaluate these other aspects of the RI role for child witnesses, providing a more holistic assessment of their efficacy.

Given the experimental nature of the research, additional methodological constraints may also have affected the findings. For example, the staged event shown to ‘witnesses’ was mild and involved the child acting as an observer rather than an active participant. Further, in practice, RIs would have had more time for discussion with those who know the witness best and more time to build rapport—and such information could have led to them using additional strategies to enable children with ASD to give best evidence. It is also possible that children with ASD were not able to identify (from the large battery of assessments administered to them) what the most salient part of the research process was (i.e., the staged event), for them to recall it one week later. In this regard, the fact that 12 of the 71 participants with ASD (17% - evenly spread across interview conditions) failed to remember anything during the Investigative Interview, as opposed to just 6 of the 199 TD participants (3%—most of them in the Best-Practice interview) is interesting to note. On the other hand, the lack of interactions between group and interview comparison for the other interviews (i.e., Verbal Labels versus Best-Practice, and Sketch-RC versus Best-Practice) indicate that for these interview comparisons, recall of correct details did not differ between children with and without ASD. Taken together, the findings do not support *overall* differences in Investigative Interview performance between children with and without ASD; rather, they emphasise that children with ASD did not experience the same improvements to recall that TD children did in RI interviews. Although previous studies reported lower recall levels in children with ASD (Bruck et al. 2007; McCrory et al. 2007), they used scripted (including misleading and specific) questions. The absence of group differences here, except in RI interviews, may imply that other types of best-practice investigative interviews (which emphasise free recall and follow-up questions based only on what the child has already mentioned) eliminate the recall disadvantage of children with ASD.

The current study offers the first empirical evidence to support the use of RIs in 6–11-year-old TD children. The implementation of the RI scheme in England and Wales is widely regarded as ground-breaking, with a range of anecdotal evidence for its effectiveness (Plotnikoff and Woolfson 2015). Further, RI schemes have international forensic relevance for criminal justice professionals in jurisdictions world-wide (see Plotnikoff and Woolfson 2015). Indeed, the findings for the TD group were powerful in showing marked improvements in the amount these children recalled in RI interviews. In this context, it is important to explore why RIs were effective for TD children. Given this was a mild event and the questioning was conducted in a familiar environment, it is likely that the pre-interview preparations (e.g., more extensive rapport building, use of calming objects) were not as important for TD participants as the within interview adaptations (e.g., simplifying the language used in the interview protocol; providing concrete aids to support verbal explanations on either the part of the interviewer or the child, if needed). As well as promoting more developmentally effective interviews, RIs could also have encouraged greater planning before the interview. Analysis of the types of information recalled in the RI condition revealed that TD children recalled significantly more people, actions, objects and general details, indicating the value of the RI condition in eliciting additional forensically *useful* information. Importantly, RIs did not encourage interviewers to prompt these areas directly; they focused on neutral follow up prompts to build on what the child had already said (as per current police practice in England and Wales; Home Office 2011).

It should be noted that there was a statistically significant difference between the amount of information elicited from TD children at Investigative Interview depending on which of the two RIs the children worked with, which held even after the characteristics of the children (e.g., age, IQ, language) were accounted for. Despite the two RIs following a standard framework within this study (agreed with a panel of four experienced RIs), RIs employ different tools and techniques in practice (as they did in the current study), some of which may be more or less effective than others. The next step will be to look at when, how and why RIs intervene during interviews; to determine which interventions are most effective, and for whom (see also O’Mahony 2012).

Regarding the other interview techniques, the Verbal Labels procedure showed the predicted beneficial effect on correct details recalled at interview without increasing incorrect items in TD children (although there was a small increase in confabulations). These results confirm and extend previous positive findings from 3- to 8-year-old TD children (Brown and Pipe 2003; Chae et al. 2014; Kulkofsky 2010) to a wider age range (up to 11 years). Further, Verbal Labels interviews significantly increased the number of setting, objects and general details recalled by TD children, which could provide key information for a case. Theoretically, the provision of external retrieval cues (Schneider 2014) is argued to elicit more detailed event information, by guiding a more exhaustive memory search and helping children to focus on forensically useful information. The ease of application and low cost of the Verbal Labels method is appealing, although further research is required to explore the increases in confabulations—these were in line with previous studies that have reported Verbal Labels interviews to increase error rates (Chae et al. 2014; Kulkofsky 2010).

Contrary to predictions, the Sketch-RC did not have a positive effect on overall interview performance in either children with ASD or TD children as per Mattison et al.’s (2015, 2016) findings (although note that Sketch-RC interviews did increase the number of objects and general details recalled by TD children). However, given the familiar location of the event (i.e., a school), the Sketch-RC procedure may not have been maximally effective. Contextual cues may be most useful if uniquely associated with an event, and unique contextual cues may have been limited in the current study. Two key methodological differences between the studies may also be relevant. First, the mean ages of Sketch-RC participants in the present research (ASD = 8 years 11 months; TD = 8 years 3 months) was slightly younger than participants in Mattison et al.’s studies (9 years 11 months). Second, the delay between viewing the event and being interviewed was around a week in the present research, but just an hour in Mattison et al.’s studies. With such different delay periods, and less mature participants, the current Sketch-RC intervention may not have been sufficiently powerful to elicit more accurate recall.

In summary, the current study found that RIs, although helpful for TD children, did not improve the volume of accurate witness recall in children with ASD. Nevertheless, ASD children did not differ from TD children when considering comparisons between Best-Practice and Verbal Labels/Sketch-RC interviews respectively. Further, children with ASD demonstrated consistently high levels of accuracy, suggesting that they can be reliable witnesses. A strength of the current study was that age and important cognitive variables were equated or controlled between interview conditions, constituting (to our knowledge) the most comprehensive approach to date in accounting for variables that may affect investigative interview performance. Further research is needed to explore the wider aspects of the RI role in more depth, particularly for children with ASD.

## Footnotes

1 In total, 22 ASD children and 165 TD children saw the event live, with the remainder (49 ASD, 34 TD) watching the event on a video. In order to assess any potential differences between live and video presentations of the event, recall at Brief interview (i.e., before allocation to investigative interview conditions) was compared for children who experienced live versus video presentations of the event. As these samples were not matched for age and IQ, both variables were used as covariates in a 2 (presentation method: Live, Video) × 2 (group: ASD, TD) independent samples ANCOVA. Error data were skewed, so log-transformations were applied. Regarding the total number of correct details recalled, there was no effect of presentation method, *F*(1, 264) = 0.43, *p* = .51, *η*_p_^2^ = 0.002, and no interaction between presentation method and group, *F*(1, 264) = 0.14, *p* = .71, *η*_p_^2^ = 0.001. There was, however, a significant effect of group, *F*(1, 264) = 19.98, *p* < .001, *η*_p_^2^ = 0.07, and both covariates were also significant (age: *F*(1, 264) = 45.78, *p* < .001, *η*_p_^2^ = 0.15; IQ: *F*(1, 264) = 25.57, *p* < .001,* η*_p_^2^ = 0.09). Similar analyses for total number of incorrect items and total confabulations revealed no significant effects. (Note: although there was a marginal effect of presentation method for incorrect items, *F*(1, 264) = 3.73, *p* = .05, *η*_p_^2^ = 0.01, the effect size was negligible, so we concluded there were no key presentation method differences).

2 In total, 136 children viewed Version ‘A’ of the staged event and 134 viewed Version ‘B’. There were no significant differences in Brief Interview performance (i.e., before allocation to interview conditions) between those who viewed the different versions of the event: Brief Interview total correct details (Mean Version A = 30.86, SD = 14.15; mean version B = 33.31, SD = 17.17; *t*(257) = 1.28, *p* = .20); Brief Interview total incorrect details (mean version A = 2.50, SD = 2.02; mean version B = 2.51, SD = 1.99; *t*(268) = 0.10, *p* = .92); Brief Interview total confabulations (mean version A = 1.38, SD = 2.20; Mean Version B = 1.84, SD = 3.08; *t*(268) = 0.86, *p* = .39). [Note: as error data were skewed, log-transformations were applied.]

3 The use of both a Brief and Investigative interview could be considered a form of repeated interviewing, which has traditionally been discouraged (e.g., Leichtman and Ceci 1995; Home Office 2011). However, Goodman and Quas (2008) suggest that the number of interviews is not as critical as the manner in which the interviews are conducted. Indeed, recent research suggests that well-conducted supplementary interviews may be an effective way of gaining investigative leads from children (Waterhouse et al. 2016). The primary concern in the present investigation was ecological validity, ensuring the study was conducted in a manner that best represented a real life police investigation.
